# Supplementary material for: Distribution and Characterization of Antimicrobial Resistant Pathogens in a Pig Farm, Slaughterhouse, Meat Processing Plant, and in Retail Stores
Source: Microorganisms. 2022 Nov 14;10(11):2252. doi: 10.3390/microorganisms10112252 (PMC9693440; doi:10.3390/microorganisms10112252)
Supplement: Supplementary file 1 [file microorganisms-10-02252-s001.zip › microorganisms-1873710-supplementary.pdf]

Supplementary data table S1. Pathogenic or spoilage bacteria isolated from the pig farms, slaughterhouse, meat processing plant, and retail stores

	Farm (3 barns, *n* = 72)								Slaughterhouse (*n* = 18)						Meat processing plant (*n* = 18)						Market (*n* = 18)	
	Pig swab	Diet	Feces	Fluid manure	Floor swab	Glove	Fan swab	Water	Carcass swab	Floor swab	Glove	Cutter swab	Water	Drain	Meat swab	Floor swab	Table swab	Water	Glove	Drain	Belly swab	Neck swab
Acinetobacter baumannii																					2	
Acinetobacter guiloniae																		1				
Acinetobacter radioresistens							1															
Aeromonas bestiarum																				1		
Aeromonas eucrenophila														1								
Aeromonas hydrophila										1							1					
Aeromonas media																				1		
Aeromonas veronii										1										2		
Aeromonas viridans									2												1	
Alcalgens faecalis						1																
Bacillus galactosidlyticus																						
Buttiauxella gaviniae									5													
Citrobacter braakii										1	1	2										
Citrobacter freundii			1								1	1								1		
Citrobacter koseri											1	2										
Corynebacterium stationis					2																	
E. coli O157:H7			1						1		1										9	
Enterobacter amnigenus					1									1								
Enterobacter asburiae		1					1															
Enteriobacter cloacae		3														1						
Escherichia coli			7	1													2					1
Escherichia hermannii	1						1															
Ewingella americana																					1	
Exiguobacterium aurantiacum												1										
Exiguobacterium sp.																6						
Hafnia alvei									2						5							3
Kocuria rhizophila																					1	
Leclercia adecarboxylata	4																					
Listeria grayi																				1		
Listeria monocytogenes															1							
Lysinibacillus fusiformis	1																				1	

Microbacterium liquefaciens													1						1		
Morganella morganii				1																	
Pantoea agglomerans								1			1									3	1
Proteus mirabilis																				5	
Proteus vulgaris	7		2																	2	
Providencia rustigianii																					1
Pseudomonas antarctica																				3	
Pseudomonas cedrina								1												1	
Pseudomonas chlororaphis									1												
Pseudomonas extremorientalis							1														
Pseudomonas fragi								1												11	1
Pseudomonas koreensis															1						
Pseudomonas libanensis							2													5	
Pseudomonas lundensis								1							3					5	20
Pseudomonas monteilii		1																			
Pseudomonas putida										1											
Pseudomonas rhodesiae																			1		
Pseudomonas synxatha																				1	
Pseudomonas taetrolens								2							2					3	
Pseudomonas tolaasii																					3
Rahnella aquatilis														1							
Raoultella ornithinolytica								1		1											
Rothia nasimurium					1																
Serratia fonticola														1							
Serratia liquefaciens								3			1		2	4	1	1		2	2	4	8
Shewanella putrefaciens										1											
Staphylococcus aureus																1					
Staphylococcus chromogenes	2																				
Staphylococcus epidermidis								1													
Staphylococcus hyicus	3																				
Staphylococcus lentus		1																			
Staphylococcus saprophyticus	4							1								1		5			1
Staphylococcus sciuri		1				1				1						17		1			1
Staphylococcus simulans	3																				
Staphylococcus warneri								1													
Yersinia enterocolitica								6		2	1									1	
